# MRONJ of the Mandible—From Decortication to a Complex Jaw Reconstruction Using a CAD/CAM-Guided Bilateral Scapula Flap

**DOI:** 10.3390/medicina59030535

**Published:** 2023-03-09

**Authors:** Robin Kasper, Mario Scheurer, Sebastian Pietzka, Andreas Sakkas, Alexander Schramm, Frank Wilde, Marcel Ebeling

**Affiliations:** 1 Department of Oral and Plastic Maxillofacial Surgery, Military Hospital Ulm, Academic Hospital of the University of Ulm, 89081 Ulm, Germany; 2 Department of Oral and Maxillofacial Surgery, University Hospital Ulm, 89081 Ulm, Germany

**Keywords:** MRONJ, mandibular reconstruction, free flap, CAD/CAM, PSI

## Abstract

Medication-related osteonecrosis of the jaw (MRONJ) has been an integral part of the maxillofacial patient population for some time. The therapeutic concept ranges from conservative approaches over less extended decortications to major jaw resections, which can result in a considerable loss of quality of life. Based on three case reports, this paper presents the long-term history of patients with MRONJ of the mandible, whose disease ultimately resulted in partial or total mandibular resection and subsequent multisegmental reconstruction using a microvascular anastomosed bone flap. Furthermore, a suitable alternative for complex mandibular reconstruction is demonstrated when using a free fibula flap is not possible. The options are limited, particularly when multisegmental restoration of mandibular continuity is required. One case presents a mandible reconstruction using a CAD/CAM-guided bilateral scapular free flap (CAD/CAM = Computer-Aided Design and Manufacturing), which has not been described for this purpose before. Due to the complexity, computer-assisted surgery and patient-specific implants seem reasonable, which is why a special focus was applied to this topic.

## 1. Introduction

Almost 20 years have passed since the first description of a correlation between the use of bisphosphonates and osteonecrosis of the jaw [1]. This side effect of bisphosphonates and other known antiresorptive drugs, such as the RANKL antibody denosumab, has become a daily routine in oral and maxillofacial surgery. It is known as medication-related osteonecrosis of the jaw (MRONJ) or antiresorptive-related osteonecrosis of the jaw (ARONJ). Other drugs that show a significantly higher prevalence of MRONJ in combination with bisphosphonates than bisphosphonates alone are the tyrosine kinase inhibitor sunitinib or the angiogenesis inhibitor bevacizumab [2,3]. Indications of antiresorptive drugs are mainly osseous metastases, osteoporosis, or malignancies of the hematopoietic system, such as multiple myeloma [4,5,6]. In addition to the primary disease, they cause a further reduction of the remaining quality of life. The cause is defined as a still incompletely understood multifactorial genesis. Besides suppression of bone turnover via osteoclast inhibition, anti-angiogenic effects, an impaired immune response, and direct tissue toxicity are discussed. Possibly crucial for the preliminary affection of the jawbone is the indirect contact of the bone with the bacterial milieu of the oral cavity via the periodontium [7,8,9,10,11,12].

The field of drug-associated osteonecrosis of the jaw is still being intensively researched to improve conservative, non-surgical therapy strategies in particular. Approaches include increasing bone remodeling as well as improving bone perfusion. Examples are the use of pentoxifylline and vitamin E, teriparatide, or hyperbaric oxygen therapy [13,14].

The disease challenges the skills of dentists and maxillofacial surgeons alike. Unfortunately, improperly performed tooth extractions or other dental surgical procedures under antiresorptive therapy continue to occur. An insufficient anamnesis may result in a long-lasting treatment with sometimes serious consequences for the patient. Based on three patient cases, this series reports on the protracted therapy of antiresorptive-related mandibular necrosis, which ranged from basic decortication to partial jaw resection following reconstruction with microvascular anastomosed bone grafts. In addition to the basic principles of MRONJ therapy, the focus is put on the possibilities of complex reconstruction of parts of the mandible using suitable osseous flaps, including computer-assisted planning and implementation.

## 2. Materials and Methods

The therapeutic consequences of refractory drug-related osteonecrosis of the mandible, based on the history of three patients treated in the department at the Military Hospital Ulm between 2008 and 2022, are demonstrated.

## 3. Patient Cases

### 3.1. Case 1

**Figure 1:**
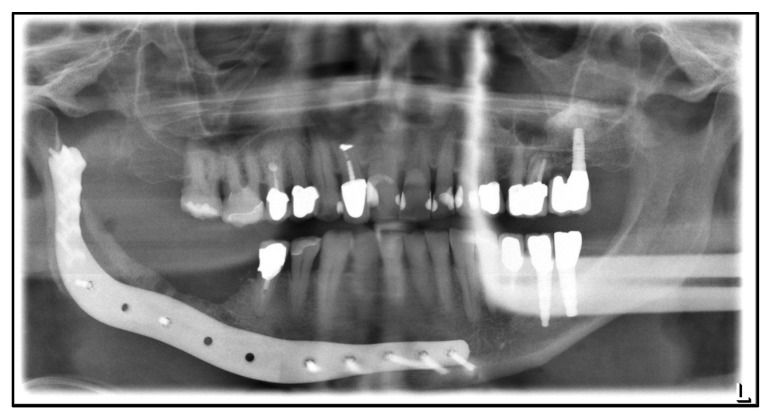
Dental X-ray after mandibular box resection and protective osteosynthesis, July 2014.

The first case involves an 81-year-old Caucasian male patient who has been under treatment for MRONJ since 2014. He was first diagnosed with prostate cancer in 2000. Besides this, he is diagnosed with arterial hypertension and hypothyroidism after hemithyroidectomy. Because of osseous metastases, zoledronate, and later denosumab, were administered. The first presentation was with an MRONJ of the right mandible. Table 1 shows the patient’s treatment course in a timeline. In 2014, a right mandibular box resection with protective osteosynthesis using a patient-specific reconstruction plate followed (Figure 1).

**Figure 2:**
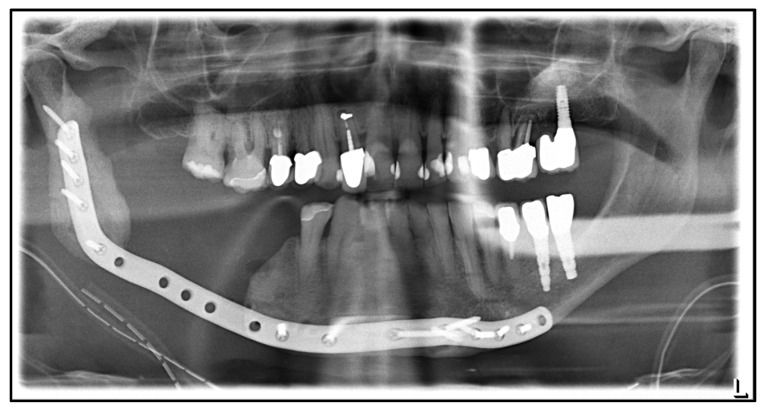
Dental X-ray after mandibular continuity resection and osteosynthesis with a patient-specific plate, August 2018.

In 2017, a reosteosynthesis was performed using a conventional hand-bent reconstruction plate due to chronic fistulation. A continuity resection was done one year later because of recurrent submental fistulation. The plate was replaced again by a patient-specific plate (Figure 2).

**Figure 3:**
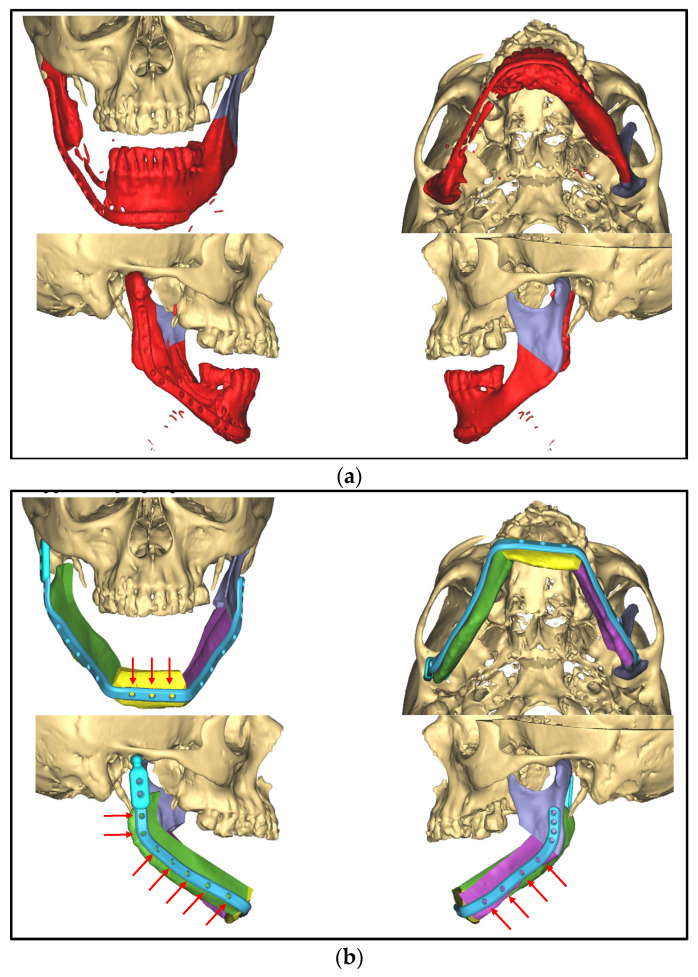

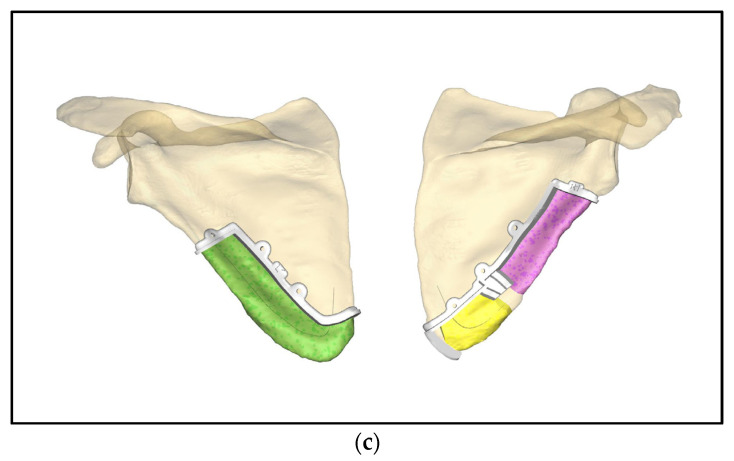
CAD/CAM-guided planning of partial mandibular resection and primary bony reconstruction using free scapular flaps from both sides: (**a**) resected mandible indicated in red; (**b**) reconstructed mandible using three segments from the right (yellow and purple) and left scapula (green), patient-specific plate with an artificial joint on the right side indicated in blue, red arrows indicate the screw positions; (**c**) posterior view on the bone harvested from the left scapula (green, 1 segment) and the right scapula (yellow and purple, 2 segments) with surgical guides.

Three months later, due to a cervical abscess, partial removal of the plate was performed. In the following years, the patient developed further multiple abscesses, whereupon in July 2022, the decision was made to perform a radical resection from the right mandible, including the temporomandibular joint to the left ramus followed by a primary bony reconstruction with a free fibula graft. A CT angiography of the pelvic and leg region was performed to evaluate the vascular supply of the lower leg. Here, a peripheral arterial disease with only a 1-vessel supply of the right and a 2-vessel supply of the left lower leg was found, which led to the decision to reconstruct the defect using a scapular flap with a skin island from both sides. This required the recontouring of the mandible with three bony segments for functional and esthetic rehabilitation. The planning was performed using CAD/CAM technique (Computer-Aided Design and Manufacturing). In addition to fabricating a patient-specific reconstruction plate with an artificial joint on the right side, surgical guides, the resection of the mandible, and the osteotomy of the scapula bone were also planned virtually and executed via cutting guides (Figure 3).

**Figure 4:**
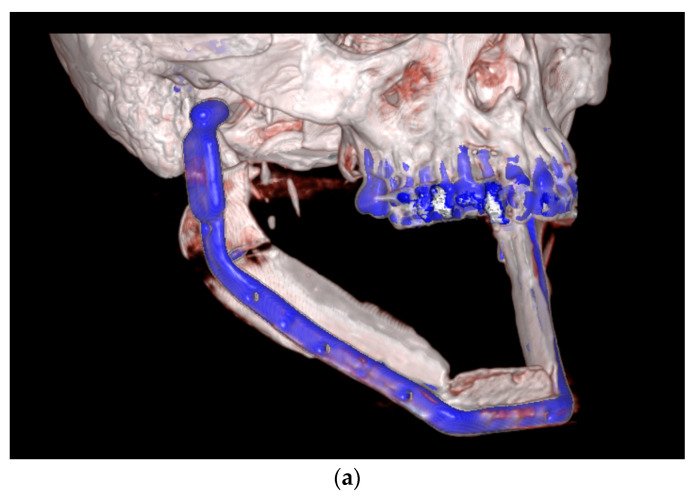

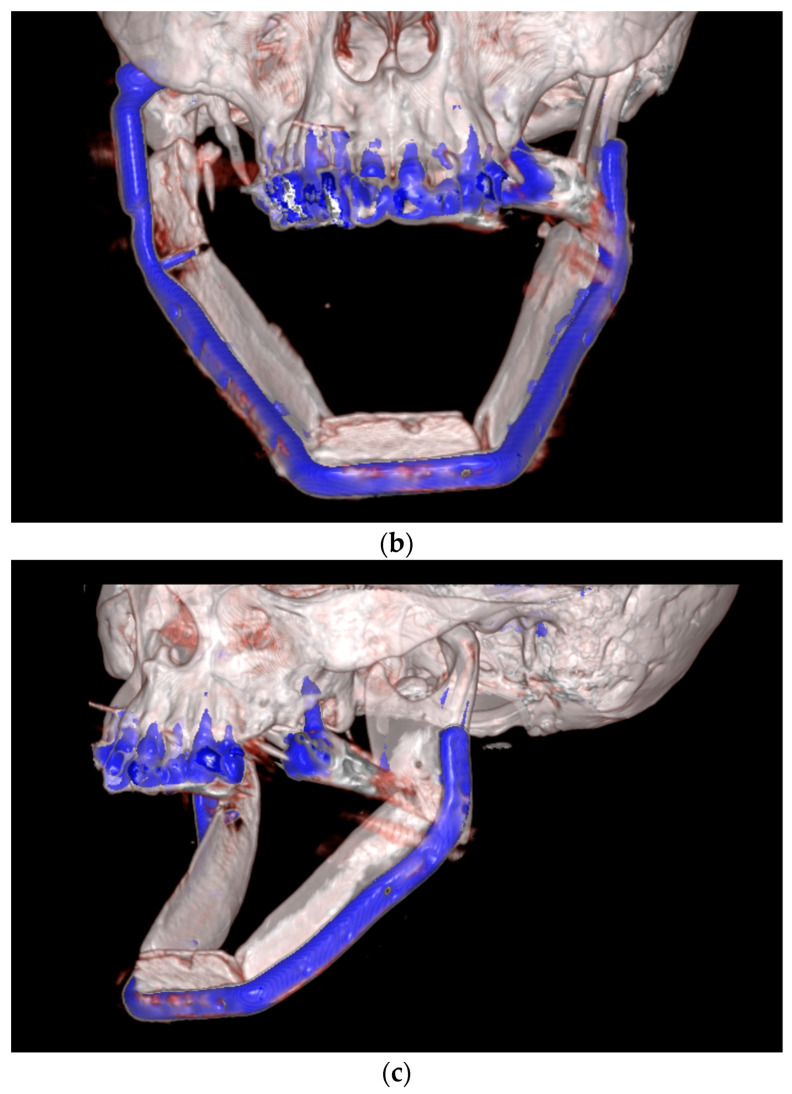
(**a**–**c**) shows the 3D reconstruction of the postoperative CT scan after resection and reconstruction of the mandible using free scapular flaps from both sides as virtually planned. The patient-specific implant is indicated in blue (along with the upper teeth).

The surgery was done 2 months later (Figure 4). On the first postoperative day, insufficiency of the anastomosis occurred. A revision was performed. In the course, necrosis of a small area of the grafted skin island developed, which could be debrided and left for secondary healing. A temporary tracheostomy was performed on the fifth postoperative day due to increased swelling. The following recovery process was free of complications. To the author’s knowledge, this is the first bilateral scapular flap that has been reported until now. Both the esthetic and functional outcomes 3 months postoperatively were satisfactory. There were no relevant mobility impairments in the donor region. Dental rehabilitation by means of implants in the neo-mandible is now planned.

### 3.2. Case 2

**Figure 5:**
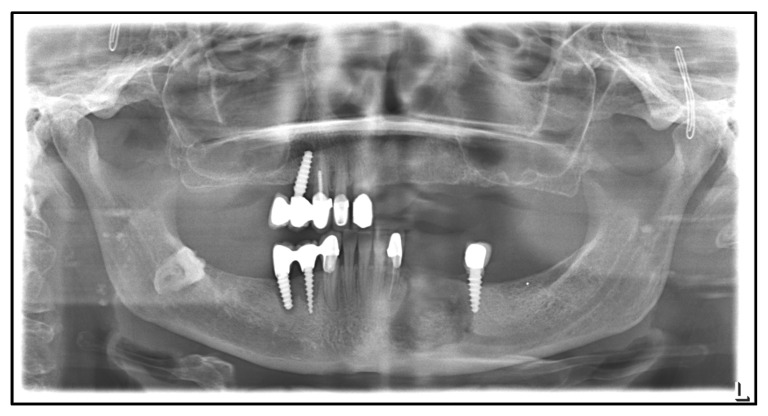
Dental X-ray at the time of admission with extended MRONJ of the left anterior mandible and ongoing intraoral fistulation, January 2020.

The second case reports a 69-year-old Caucasian female patient who first presented to the clinic in January 2020 on the referral of an oral surgeon with drug-associated mandibular necrosis in the left premolar region. Four years earlier, osseous metastatic breast cancer was diagnosed. Tumor resection was performed with adjuvant radiation and intravenous bisphosphonate therapy with zoledronate 4 mg every 6 months for approximately 3 years. In 2019, the patient first noticed a pus leakage in the area of the lower left premolars. After ineffective conservative therapy, the general dentist performed the removal of the lower left canine and first premolar under antibiotic prophylaxis. As pus continued to leak, the patient was referred to an oral surgeon’s consultation, who finally sent the patient to the hospital (Figure 5).

**Figure 6:**
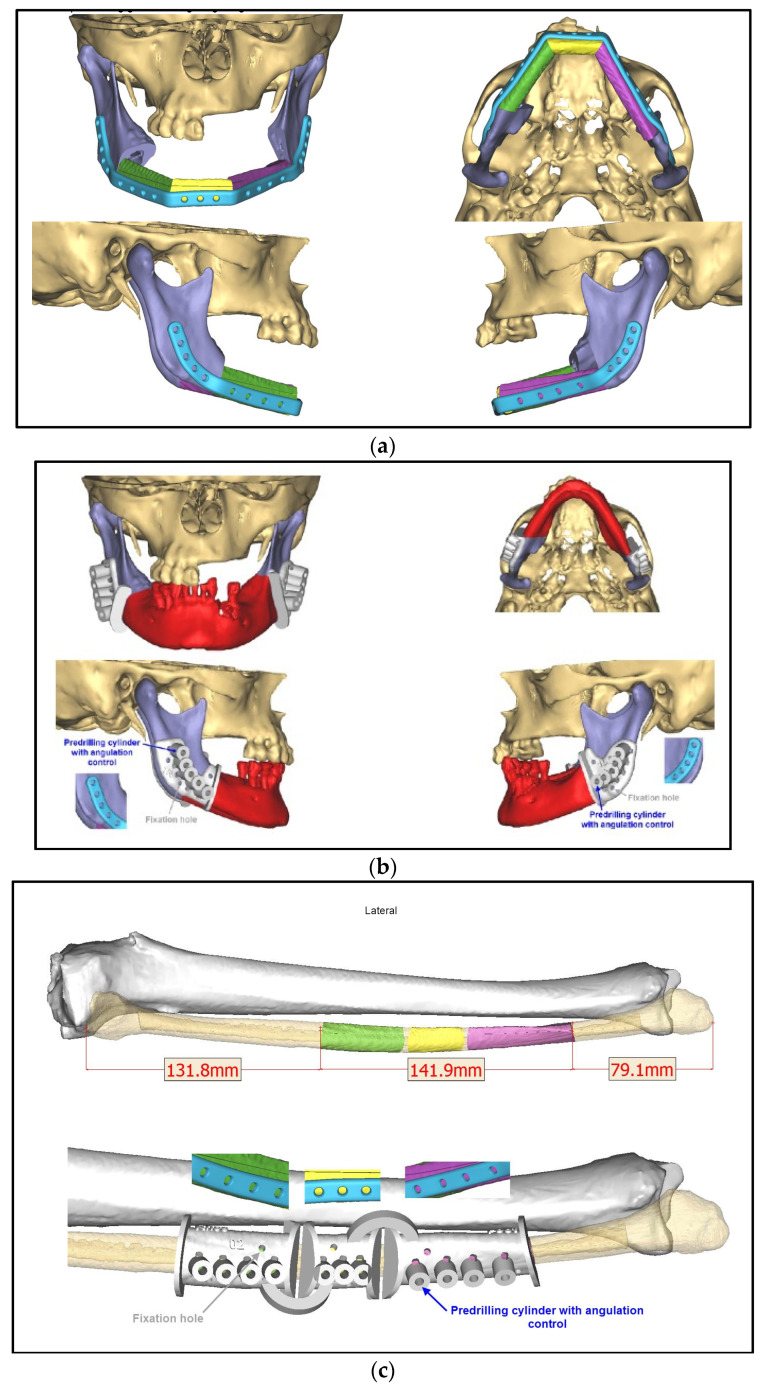
CAD/CAM-guided planning of the resection and reconstruction of the mandible using a free fibular flap: (**a**) planned postoperative situation after resection and reconstruction with three fibula segments and patient-specific plate; (**b**) cutting-/drill-guides for resection of the mandible (red) and pre-drilling of the holes for fixation of the patient-specific reconstruction plate (**c**) patient’s right fibula with colored osteotomy segments for the planned reconstruction of the mandible; surgical guides for osteotomy of the fibula.

**Figure 7:**
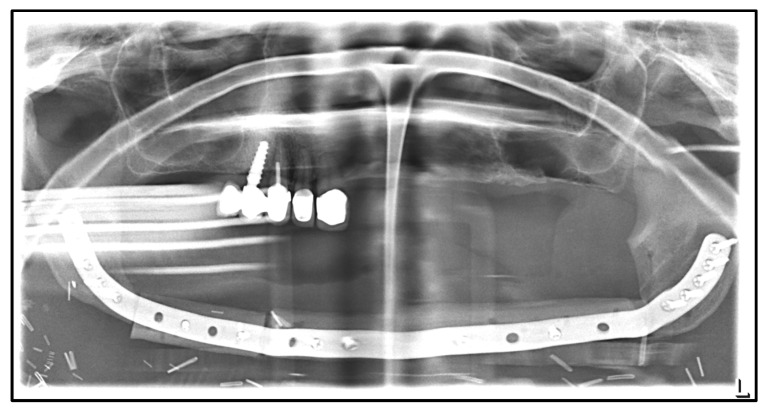
Dental X-ray after mandibular resection and primary bony reconstruction using a free fibular flap, April 2020.

Table 2 shows the patient’s treatment course in a timeline. Initial findings showed an inflammatory altered mucosa in the described region with probing depths of 7 mm. Three-dimensional imaging revealed extended osteolysis of the mandible with sequestration. Due to the size of the defect, CAD/CAM-guided planning of a partial mandibular resection with continuity defect from the right to the left jaw angle was performed (Figure 6). In April 2020, resection of the mandible and primary reconstruction of the defect using a free fibula flap and a patient-specific reconstruction plate followed (Figure 7).

**Figure 8:**
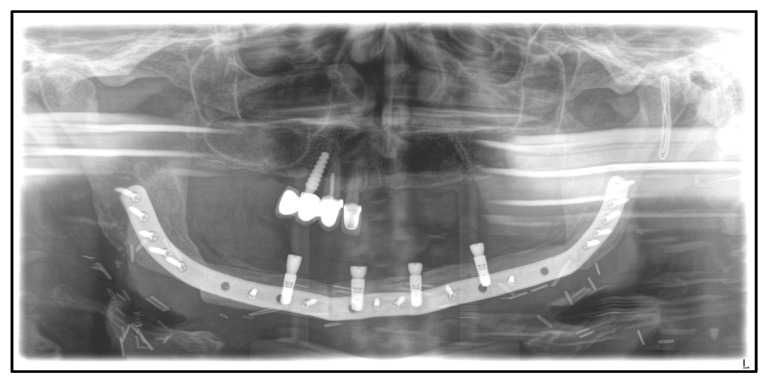
Dental X-ray after implantation of four endosseous implants in the neo-mandible for dental rehabilitation and extraction of the upper first incisor on the right, August 2021.

Furthermore, a temporary tracheostomy was needed. On the fourth postoperative day, an increase in infection parameters with fever and a decrease in oxygen saturation was observed. The chest X-ray showed bilateral pericardial infiltrates. Antibiotic therapy with piperacillin and tazobactam 4.5 g for a total of 8 days was given due to suspected aspiration pneumonia. This led to a decrease in infection parameters and symptoms. After the closure of the tracheostomy on the 13th postoperative day, the patient was discharged for further outpatient treatment. The follow-up was without any complications. Because of the patient’s wish for dental rehabilitation in the mandible, computer-assisted placement of four implants in the neo-mandible was planned and performed 12 months postoperatively. Four months later, the first upper right incisor was extracted due to decay following the smoothing of bony edges and primary wound closure (Figure 8). The upper jaw is currently restored with a removable partial denture.

### 3.3. Case 3

**Figure 9:**
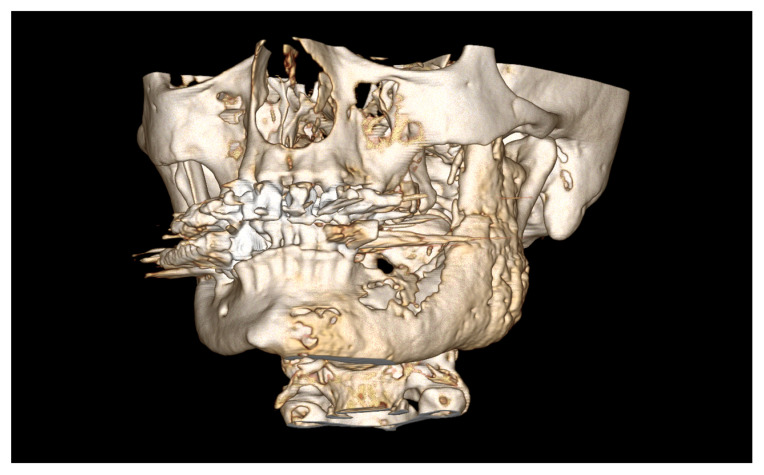
CT reconstruction of the midface and mandible shows a massive periost reaction due to MRONJ of the mandible.

The final case describes a rare complication of an MRONJ patient after total mandibular resection and reconstruction using a free fibula flap. The 67-year-old woman presented to the hospital with an extended MRONJ in the mandibular region under bisphosphonate therapy for osseous metastases from breast cancer (Figure 9).

**Figure 10:**
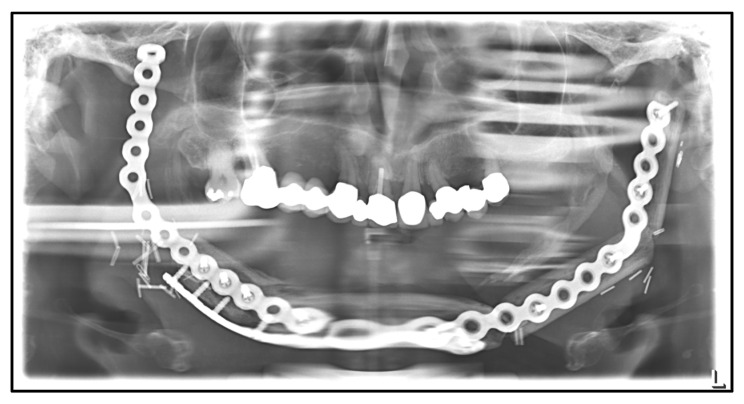
Dental X-ray after total mandibular resection and reconstruction with a fibular free flap. Fractured hand-bent plate in the right anterior region and supporting reconstruction plate on the basal rim of the neo-mandible.

A large partial resection of the mandible from the left temporomandibular joint to the right jaw angle was finally performed. The defect was reconstructed by a free fibula flap. Four months later, the rest of the mandible, including the right temporomandibular joint, had to be removed as well. Three months postoperatively, a submandibular abscess occurred on the right side. Several months later, a plate fracture of the hand-bent plate occurred. The plate was left in situ to hold the fibular grafts and was supported by another reconstruction plate, which was attached to the basal rim of the neo-mandible (Figure 10).

**Figure 11:**
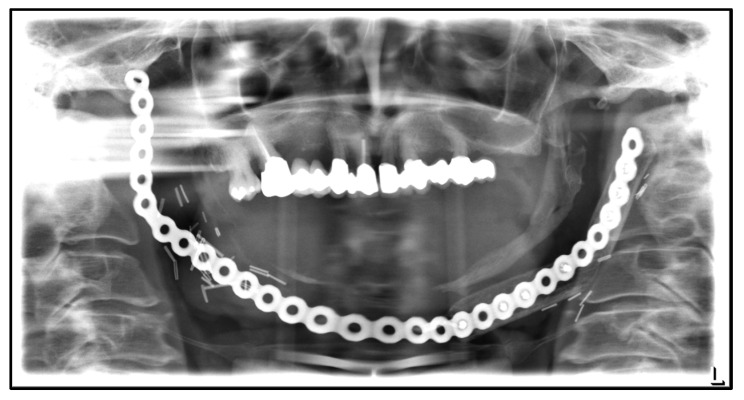
Dental X-ray after removal of the osteolytic destructed lateral right and anterior fibula segment and following re-osteosynthesis due to chronic inflammation.

Shortly after, the lateral right and anterior fibula segments had to be removed due to osteolysis, presumably because of chronic inflammation ongoing for several years (Figure 11). In addition, the reconstruction plates were removed as well, and the mandible was reconstructed alloplastically in this area again; several submandibular fistula tracts were excised, and scar correction was performed in the neck region. The patient did not wish for another bony reconstruction. For about 10 years, the patient has been free of recurrence or other complaints, so a wait-and-see approach was decided in agreement with the patient. The mandible was not prosthetically rehabilitated due to the lack of underlying bone.

## 4. Discussion

Medication-related osteonecrosis of the jaw presents immense challenges not only to the oral and maxillofacial surgeon but also to the patient. Therefore, the full competence and interdisciplinary cooperation of oncologists, dentists, and oral and maxillofacial surgeons are a key requirement for not only early detection and sufficient treatment but also for the prevention of its development.

Therefore, the therapy of antiresorptive-induced osteonecrosis begins with its prophylaxis, meaning interventions prior to the administration of antiresorptive drugs. In case of needed antiresorptive therapy, besides informing the patient about the risk of developing an MRNOJ (risk profile assessment depends on the agent, frequency and duration of administration, dosage, as well as the application form of antiresorptives), a focused screening with the elimination of possible bacterial entry sites should be performed [15,16,17,18,19,20,21,22,23,24,25,26,27,28,29,30]. The indication for surgical procedures on the jaw should be made carefully under or after the administration of antiresorptive drugs. If surgical intervention is indicated, preventive steps are required to avoid the development of an MRONJ. These include perioperative systemic antibiotics, smoothing of sharp bony edges, primary wound closure, and a close follow-up for at least 4–6 weeks [31,32,33,34,35,36]. In the case of a present MRONJ, surgical therapy is indicated, especially in patients with extended or multiple lesions. Most important is a total resection of the necrotic bone with a modeling osteotomy, primary wound closure, and a prolonged administration of perioperative systemic antibiotics. Additionally, inpatient care, intravenous application of antibiotics, treatment under general anesthesia, or postoperative tube feeding may be necessary. Conservative therapy with systemic antibiotics and antimicrobial mouth rinses can be considered for small and asymptomatic lesions or for symptom control, especially in patients with a poor general condition or advanced cancer, after careful evaluation and in agreement with the patient [37,38,39,40,41,42]. However, in the case of extended necrosis of the mandible, it may be necessary to perform a partial resection leading to a continuity defect, which then can be reconstructed alloplastically using a load-bearing osteosynthesis plate alone or with a microvascular anastomosed bone graft. When using CAD/CAM technology for planning extensive resections of the mandible, a critical point is the determination of the resection margins. The extent of the affected bone is often much higher than it clinically appears and can usually only be adequately assessed intraoperatively. Therefore, determining the margins should be planned carefully, considering the clinical and radiological findings. Major advantages of computer-assisted planning are the feasible preservation of the relations between the upper and lower jaw, including the temporomandibular joint’s position and the optimal positioning of the bone graft for later implant placement. These points are particularly crucial for multisegmental reconstructions of the mandible. In the maxilla, besides bony reconstruction, local flap techniques or defect coverage with obturator prostheses are possible [41,43,44].

The defects of the described cases after resection required a bony reconstruction of the mandible. This should be done not only for aesthetic reasons to avoid the image of a so-called “Andy Gump deformity” but also to restore sufficient swallowing, speaking, and chewing function [45,46]. Alloplastic reconstruction of wide-span defects significantly increases the risk of complications such as extraoral plate exposure or fracture compared to smaller defects [47,48,49]. Moreover, alloplastic reconstructions show an increased rate of so-called hardware-associated complications, such as loosening or fracture of plates and screws, compared to bony reconstructions [50,51,52]. The first choice is usually the fibular flap. It is the longest bone flap that can be taken and is, therefore, ideal for replacing two or more mandibular segments. Furthermore, a skin flap can be harvested for intraoral or extraoral defect coverage [53]. Despite the relatively low height compared to the natural mandible, the fibula is very well suited for following dental rehabilitation by endosseous implants. These attain high primary stability due to the high amount of cortical bone [54,55]. A disadvantage is the high variability of the perforator vessel for the skin island, which causes a loss of the skin flap in approximately 7% of the cases [56]. The morbidity of the donor site is comparatively low. Relevant stability or movement restrictions are not to be expected. Complications such as hypesthesia of the lateral malleolus or edema are rare [57,58,59,60]. Certainly, the most important point to be considered preoperatively is a sufficient blood supply of the lower leg. If this requirement of the donor region is not given due to peripheral arterial disease or chronic venous insufficiency, an alternative graft must be used. Besides the free iliac crest graft, the scapular flap can be used as done in the first case. Advantage of this graft is the generally stable vascular supply with little arteriosclerosis and mostly hairless skin. The main disadvantage compared to the fibula is the limited amount of bone available, which is why the scapula from both sides had to be used due to the large defect. This circumstance aggravates the second important disadvantage of the scapular graft. Compared to the lifting of a fibular graft, a two-team approach is not possible. In this case, the patient had to be repositioned and draped again four times, considerably increasing the surgery duration. Another disadvantage is the blade-like geometry of the scapula, which makes dental rehabilitation by implant placement in the neo-mandible difficult. However, implant placement is possible in principle [61].

The insertion of dental implants into a fibular flap has become common practice and is well-studied. The success rate is high, about 98%, after 40 months [62]. As described earlier, the fibula, with its high amount of cortical bone, is ideal for good primary implant stability. Another significant advantage is the direct vascularization of the bone. Chiapasco et al. describe reduced bone resorption compared to non-vascularized grafts [63]. Therefore, free bone flaps are further suitable for primary implantation due to the direct blood supply after connection in the recipient site [64]. However, it must be considered that, depending on the procedure, there may be an extended ischemia time, which in turn increases the risk of damage or loss of the graft. Therefore, especially in primary implantation, a CAD/CAM-assisted approach is reasonable considering the variable anatomy of the fibula, the existing reconstruction plate with associated screws, and the aim for a good functional result [65]. In any case, the success rate of primarily placed implants compared with secondarily placed implants seems comparable [62,66]. Currently, we have no data concerning the difference in osseointegration of dental implants in free fibular grafts in MRONJ patients compared to fibula flaps where no antiresorptive medication was given. Regarding the natural mandible, there seem to be no significant differences in dental implant survival in MRONJ patients compared to healthy patients [67,68,69]. Last, the long-term resorption of fibula grafts is significantly lower than in the natural mandible, whether dentate or edentulous [70,71].

The risk of long-term graft loss due to atrophy or resorption is therefore considered to be rather low. However, in the third case, osteolysis of two fibula segments occurred after approximately 3 years, requiring the removal of the affected sections. The cause was probably a chronic inflammatory reaction. Mertens et al. describe a resorption rate of about 5% after 6 months, 8% after 11 months, and 17% after 17 months [72]. Factors leading to an increased resorption rate include female gender, a high number of osteotomies of the fibula, and injury to the artery supplying the bone marrow [73,74].

## 5. Conclusions

MRONJ has become an integral part of the daily routine in maxillofacial surgery and often ends up in the long-term treatment of the patients. In the author’s opinion, the causes are the continued insufficient education of patients treated with antiresorptives due to low awareness of the treating orthopedist or oncologist, but also the dentist. Essential for prevention is a strict indication for general surgical interventions and specific steps to be taken in case of unavoidable surgery. Most importantly, this includes tooth extractions, which should be performed under antibiotic protection with smoothing of bony edges and adequate primary wound closure.

If MRONJ is present, admission to a clinical facility should be made at an early stage. When the surgical options are exhausted by necrotomies in terms of decortications of the affected bone, the radical resection of larger parts of the jaw is inevitable. In the mandible, this is usually associated with a continuity resection. In the long term, the defect should be restored by osseous reconstruction as alloplastic reconstruction alone often results in extraoral plate exposure or fatigue fracture of the plate. The free fibula flap is the method of choice. Advantages are the adequate amount of bone for reconstruction of large mandibular sections, the low donor site morbidity, and an excellent suitability for dental rehabilitation.

The bilateral scapula flap proved to be a good alternative to the fibula and iliac crest flap. The basic advantages of this flap are mostly stable vascular supply with little arteriosclerosis, hairless skin with a small amount of subcutaneous fatty tissue, and the mostly successful direct wound closure of the donor site. The disadvantage is mainly the considerably higher time required for the procedure. Due to anatomical conditions, a two-team approach is not possible. Furthermore, the partly blade-like geometry and the bone architecture of the scapula are often less suitable for dental rehabilitation using implants in the neo-mandible.

## Figures and Tables

**Table 1:** Timeline of patient case no. 1, PSI = patient-specific implant, CAD/CAM = Computer-Aided Design and Manufacturing.

Date	Intervention
2000:	Initial diagnosis of prostate cancer with vertebral metastasis. Following radical prostatectomy with adjuvant radiotherapy and intravenous bisphosphonate therapy with zoledronate 4mg monthly and denosumab every 6 weeks.
July 2014:	Diagnosis of MRONJ in the right molar region of the mandible. Following mandibular box resection and protective plate osteosynthesis with a patient-specific plate.
August 2016:	Extraction of the second lower right premolar with smoothening of bony edges due to extraoral chronic fistula.
June 2017:	Removal of the reconstruction plate in the right mandible, re-osteosynthesis, and excision of a submandibular fistula on the right.
August 2018:	Partial mandibular resection with continuity defect and alloplastic reconstruction using a patient-specific plate, excision of a submental fistula
November 2018:	Cervical abscess on the right side with infected osteosynthesis material. Decortication and partial removal of the PSI.
December 2018:	Submental abscess on the left: extraoral abscess incision.
April 2019:	Cervical abscess in the right jaw angle with extraoral plate exposure and chronic-purulent fistula: extraoral abscess incision.
February 2022:	Paramandibular abscess on the left: intraoral abscess incision.
July 2022:	Submandibular abscess on the left: extraoral abscess incision.
September 2022:	Partial mandibular resection from the left mandibular angle to the right mandible, including the right temporomandibular joint, CAD/CAM-guided reconstruction with a bilateral free scapula flap with skin island, and patient-specific plate. In the course: - revision of the vascular anastomosis of the left scapula flap; - temporary tracheotomy due to swelling; - debridement of necrotic sections of the skin graft.

**Table 2:** Timeline of patient case no. 2, PSI = patient-specific implant, CAD/CAM = Computer-Aided Design and Manufacturing.

Date	Intervention
2016:	Diagnosis of breast cancer with osseous metastasis. Mammectomy, adjuvant radiation, and intravenous bisphosphonate therapy with zoledronate 4 mg every 6 months (2016–January 2019).
2019:	Extraction of the lower left canine and first premolar due to intraoral pus leakage by the general dentist.
January 2020:	Admission to hospital with MRONJ of the left mandible and recurrent fistulation/pus leakage.
April 2020:	Partial mandibular resection from the left to the right mandibular angle, CAD/CAM-assisted reconstruction using a free fibular graft and PSI, temporary tracheostomy. In the course: development of aspiration pneumonia treated with piperacillin and tazobactam 4.5 g for 8 days.
April 2021:	Placement of four dental implants in the neo-mandible for dental rehabilitation.
August 2021:	Surgical removal of the upper right incisor, smoothening of the bone, primary wound closure, perioperative antibiosis with ampicillin and sulbactam

## Data Availability

Data is contained within the article.
